# A retrospective analysis of specialty match rate and gender trends in Canadian residency applications (2019–2024)

**DOI:** 10.1371/journal.pone.0334134

**Published:** 2025-10-30

**Authors:** Lucy Hui, Katherine Feng, Jody Tao, Oswin Chang

**Affiliations:** Faculty of Medicine, The University of British Columbia, Vancouver, BC, Canada; NYU Grossman School of Medicine: New York University School of Medicine, UNITED STATES OF AMERICA

## Abstract

**Background:**

This study examines Canadian medical graduate (CMG) match outcomes from 2019 to 2024, focusing on applicant numbers, gender distribution, and match success.

**Methods:**

A retrospective analysis was conducted using publicly available data from the CaRMS match reports. Specialty-specific application numbers and first-choice match rates were extracted. Match rates were calculated as the number of matriculates divided by the number of applicants, while competitiveness was determined by the number of first-choice applications per available position. Specialties were categorized into clinical, surgical, and diagnostic disciplines for trend analysis.

**Results:**

From 2019 to 2024, CMG applicants decreased slightly from 5380 to 5346, while the total quota increased from 2800 to 2918. Family medicine saw a significant decrease in applications (r² = −0.849, p = 0.03), while anesthesiology had a significant increase (r² = 0.950, p < 0.01). Diagnostic disciplines like neuropathology decreased significantly (r² = −0.887, p = 0.02), while diagnostic radiology increased (r² = 0.842, p = 0.03). Surgical disciplines, including plastic surgery, had steady increases, with vascular surgery doubling its applications by 2023. Female applicants increased in clinical and surgical specialties but decreased in diagnostics. Match rates improved in family medicine (r2 = 0.964, p < 0.01), medical genetics (r2 = 0.817, p = 0.04), psychiatry (r2 = 0.839, p = 0.04), public health (r2 = 0.939, p < 0.01), and diagnostic and clinical pathology (r2 = 0.850, p = 0.03), while diagnostic radiology (r2 = −0.825, p = 0.04) declined. Female applicants had higher match rates in ophthalmology and pediatric neurology, while males led in orthopedic surgery.

**Conclusion:**

Shifts in Canadian residency match trends from 2019 to 2024 may reflect the impact of the COVID-19 pandemic and evolving societal priorities. Ongoing monitoring of these trends is essential to ensure alignment with healthcare needs.

## 1. Introduction

The Canadian Resident Matching Service (CaRMS) administers the process of matching medical trainees to residency positions across Canada and operates four services: the main residency (R-1) match, the medicine subspecialty match, the family medicine/enhanced skills match, and the pediatric subspecialty match [1]. The R-1 match has two phases, known as iterations. In the first iteration, graduates of Canadian and American medical schools (CMGs) and graduates of international medical schools (IMGs) are considered separately for different seats. In the second iteration, CMGs and IMGs compete for the same pool of seats that were not filled in the first iteration. Applicants are generally assessed by the admissions committees of individual programs on the basis of materials such as personal letters, reference letters, and interview performance. Admissions committees and applicants then rank their preferences and are algorithmically matched.

While a number of recent studies using CaRMS data focus on a specific specialty or area (e.g., surgical specialties), analysis of match rate and gender trends for all specialties may reveal broader patterns and has not been updated since 2019 [2]. Since 2019, notable events such as the COVID-19 pandemic [3], expansion of artificial intelligence in clinical practice [4], and the newly implemented CaRMS self-identification questionnaire providing diversity data to residency programs [5], are novel variables that may impact the CaRMS process. Additionally, systemic level changes in payment models aimed at attracting and retaining family physicians, such as British Columbia’s Longitudinal Family Physician model and Alberta’s Alternative Relationship Plans, may reveal early trends in family medicine applications [6]. Current match trends are a projection of the future supply of physicians, which may be useful information for the development of policies and incentives aimed at addressing physician shortages and distribution imbalances, both at the level of government and individual programs.

The aim of this review is therefore to explore how each specialty involved in the R-1 match has evolved between 2019 and 2024 in terms of four domains: applicants over time, proportion of applicants by gender, match outcomes, and gender differences in match outcomes. The scope of this study will be limited to the CMG stream of the first iteration R-1 match.

## 2. Methods

A retrospective analysis of a longitudinally maintained database retrieved from the Canadian Resident Matching Service (CaRMS) was conducted. CaRMS publishes its match reports annually, making them publicly accessible online. This study was exempt from ethics approval by the University of British Columbia’s Behavioural Research Ethics Board since institutional review board approval is not required for research using publicly available and de-identified data.

Data on specialty-specific applications and first-choice match rates from the past six match cycles (2019–2024) were obtained from the “Data and Reports” section of the CaRMS website [7]. This study focused exclusively on CMGs to ensure a homogenous sample and to align with the primary target population of CaRMS. Only data from the first iteration of the match were included to maintain consistency and comparability, as subsequent iterations often involve different applicant dynamics and selection criteria. Additionally, gender “X” was excluded due to its small sample size, which limited statistical reliability and prevented meaningful analysis.

This study aimed to explore four key outcomes: Q1) how the total number of applicants for each specialty has evolved over time, Q2) variations in the proportion of applicants by gender across specialties over the years, Q3) trends in match outcomes—specifically, being matched to a first-choice specialty—across different specialties, and Q4) gender differences in match outcomes within each specialty. Match rates were calculated as the number of matriculates divided by the number of applicants. Competitiveness was calculated as the number of first choice applications divided by the quota.

All residency specialties were examined and categorized into three groups for trend analysis: clinical disciplines, surgical disciplines, and diagnostic disciplines. Clinical disciplines included anesthesiology, dermatology, emergency medicine, family medicine, internal medicine, medical genetics and genomics, neurology, pediatric neurology, pediatrics, physical medicine and rehabilitation, psychiatry, public health and preventive medicine, and radiation oncology. Surgical disciplines included cardiac surgery, general surgery, neurosurgery, obstetrics and gynecology, ophthalmology, orthopedic surgery, otolaryngology, plastic surgery, urology, and vascular surgery. Diagnostic disciplines included diagnostic and clinical pathology, diagnostic and molecular pathology, diagnostic radiology, hematological pathology, medical microbiology, neuropathology, and nuclear medicine. Applicants to clinical investigator or research-track surgical positions were excluded due to both the inconsistent availability of these positions throughout the study period and the small sample size.

Pearson correlation was used to determine trends in application number. The Cochran-Armitage trend test for proportions was employed to evaluate trends in the proportion of female first-choice applicants. P values less than 0.05 were considered statistically significant. Statistical analyses were conducted using R version 4.4.2.

## 3. Results

### 3.1 Q1: How has the total number of applicants for each specialty evolved?

From 2019 to 2024, the number of CMGs who applied through CaRMS decreased from 5380 to 5346. Concurrently, from 2019 to 2024, the total quota offered to CMG applicants increased from 2800 to 2918.

**Fig 1:**
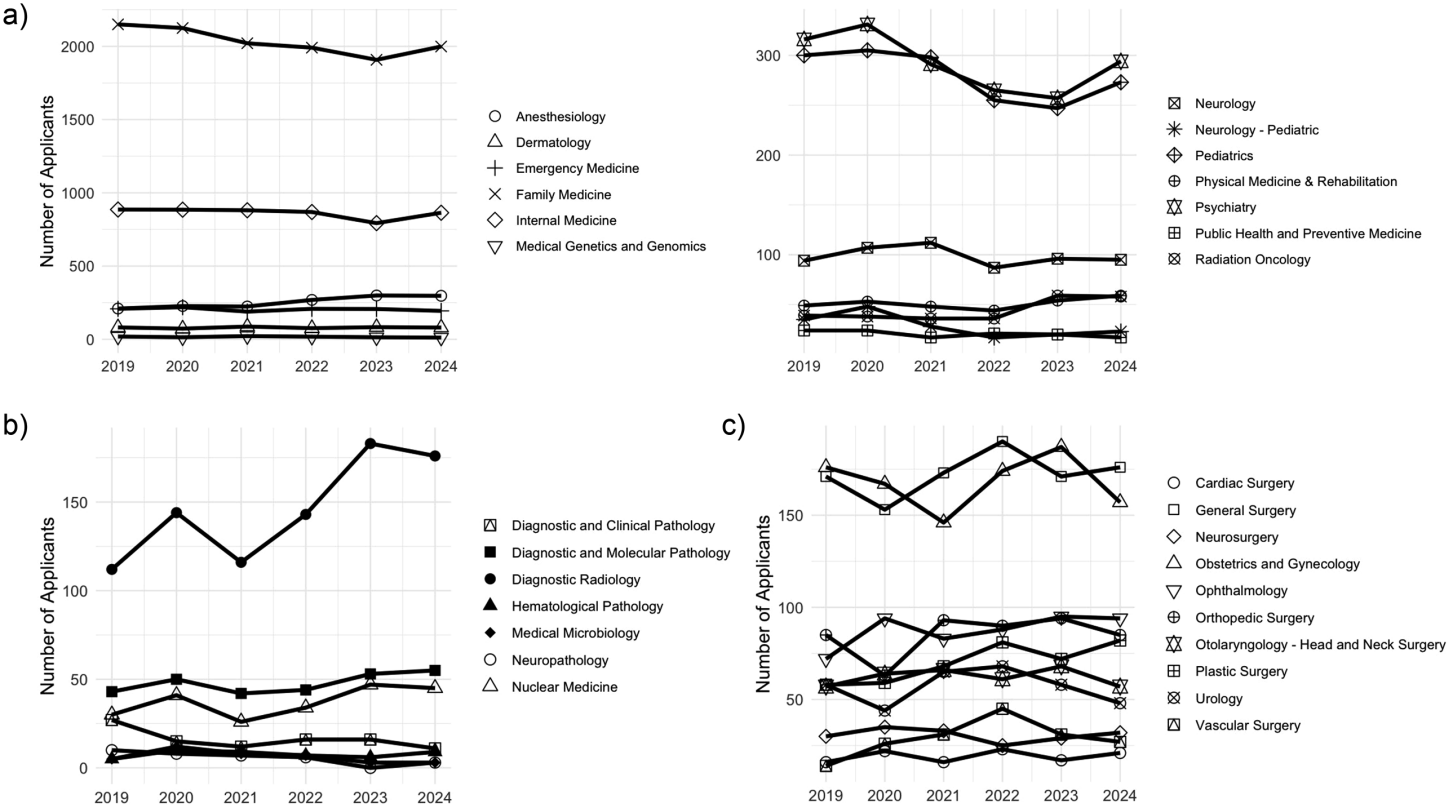
Total number of applications to each discipline grouped by (a) clinical disciplines, (b) diagnostic disciplines, and (c) surgical disciplines.

Within the clinical discipline cluster, family medicine had a significant decrease in the number of applications (r2 = −0.849, p = 0.03) and anesthesiology had a significant increase in the number of applications (r2 = 0.950, p < 0.01) from 2019 to 2024. Five disciplines (family medicine, internal medicine, psychiatry, pediatrics, medical genetics and genomics) experienced a consistent decline in the number of applications from 2020 to 2023 while only two disciplines (anesthesiology, radiation oncology) experienced a consistent increase in the number of applications. Looking at year-to-year percentage change, radiation oncology experienced the greatest increase in application number at 38.9% from 2022 to 2023, while pediatric neurology experienced the greatest decrease in application number at 71.4% from 2020 to 2021. All other disciplines exhibited a relatively consistent trend in the number of applications received (Fig 1a, S1 Table).

Within the diagnostic discipline cluster, neuropathology had a significant decrease in the number of applications (r2 = −0.887, p = 0.02) and diagnostic radiology had a significant increase in the number of applications (r2 = 0.842, p = 0.03). Three disciplines (diagnostic radiology, diagnostic and molecular pathology, nuclear medicine) experienced an overall increase in the number of applications from 2019 to 2024. Two disciplines (diagnostic and clinical pathology, neuropathology) decreased by approximately one third of applications from 2019 to 2024. All other disciplines (hematological pathology, medical microbiology) exhibited a relatively consistent trend in the number of applications received. Looking at year-to-year percentage change, hematological pathology experienced the greatest increase in application number at 58.3% from 2019 to 2020 while diagnostic and clinical pathology experienced the greatest decrease in application number at 80% from 2019 to 2020 (Fig 1b, S1 Table).

Within the surgical discipline cluster, plastic surgery had a significant increase in the number of applications (r2 = 0.886, p = 0.02). Six disciplines (general surgery, obstetrics and gynecology, plastic surgery, ophthalmology, otolaryngology, urology) experienced consistent increases in application numbers across at least a three year duration. Vascular surgery approximately doubled the number of applications from 2019 to 2023. Looking at year-to-year percentage change, vascular surgery experienced the greatest increase in application number at 46.2% from 2019 to 2020, but it also experienced the greatest decrease in application number at 45.1% from 2022 to 2023. All other specialties exhibited a relatively consistent trend in the number of applications received (Fig 1c, S1 Table).

In S1 Fig, diagnostic radiology experienced a consistent increase in competitiveness from 90% to 150% from 2019 to 2024. On average, the surgical discipline experienced a higher level of competition than other disciplines.

### 3.2 Q2: How has the proportion of applicants by gender varied across specialties overtime?

From 2019 to 2024, the number of female applicants who applied through CaRMS increased from 1587 to 1746, while the number of male applicants decreased from 1314 to 1187 (S2 Table).

**Fig 2:**
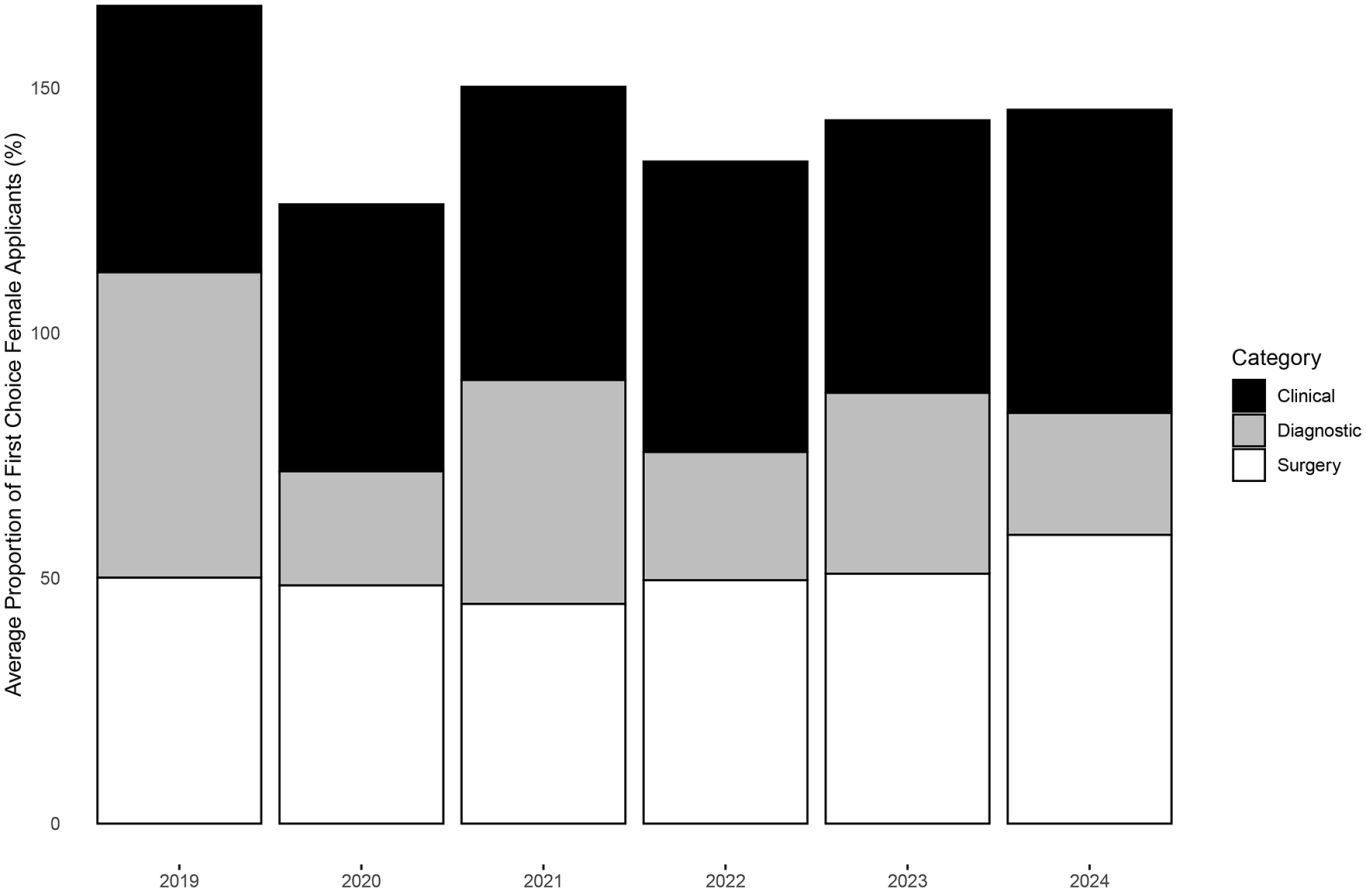
Average proportion of first choice female applicants stratified by specialty category.

In Fig 2, the average proportion of female applicants has increased overall from 2019 to 2024 in the clinical and surgical discipline clusters, while the average proportion of female applicants has decreased approximately 30% in the diagnostic cluster. However, the Cochran-Armitage trend test for proportions was insignificant across all three clusters (clinical p = 0.31, diagnostic p = 0.36, surgery p = 0.54).

Within the clinical discipline cluster, five disciplines consistently had more female applicants than male applicants (dermatology, family medicine, pediatric neurology, pediatrics, psychiatry), while one discipline consistently had more male applicants than female applicants (anesthesiology). Across all six years, approximately 80% of the applications to pediatrics were from female applicants (S2 Table).

Within the surgical discipline cluster, two disciplines consistently had more female applicants than male applicants (general surgery, obstetrics and gynecology) while three disciplines consistently had more male applicants than female applicants (neurosurgery, ophthalmology, orthopedic surgery). Across all six years, approximately 90% of the applications to obstetrics and gynecology were from female applicants. Cardiac surgery experienced the greatest percentage difference in gender representation in applications from 2023 (23.1% female) to 2024 (68.8% female). On the other hand, urology experienced approximately a 20% increase in the number of female applications from 2019 to 2024 (S2 Table).

Within the diagnostic discipline cluster, zero disciplines consistently had more female applicants than male applicants, while two disciplines consistently had more male applicants than female applicants (diagnostic radiology, nuclear medicine). Across all six years, approximately 70% of applications to diagnostic radiology and nuclear medicine were from male applicants. Gender proportions appear to be approximately stable across all years in the diagnostic discipline cluster, with the exception of diagnostic and molecular pathology in 2019 and 2021 (S2 Table).

### 3.3 Q3: What are the trends in match outcomes by specialty?

From 2019 to 2024, the total number of matriculates increased from 2800 to 2918. However, the number of applications decreased from 5380 to 5346.

**Fig 3:**
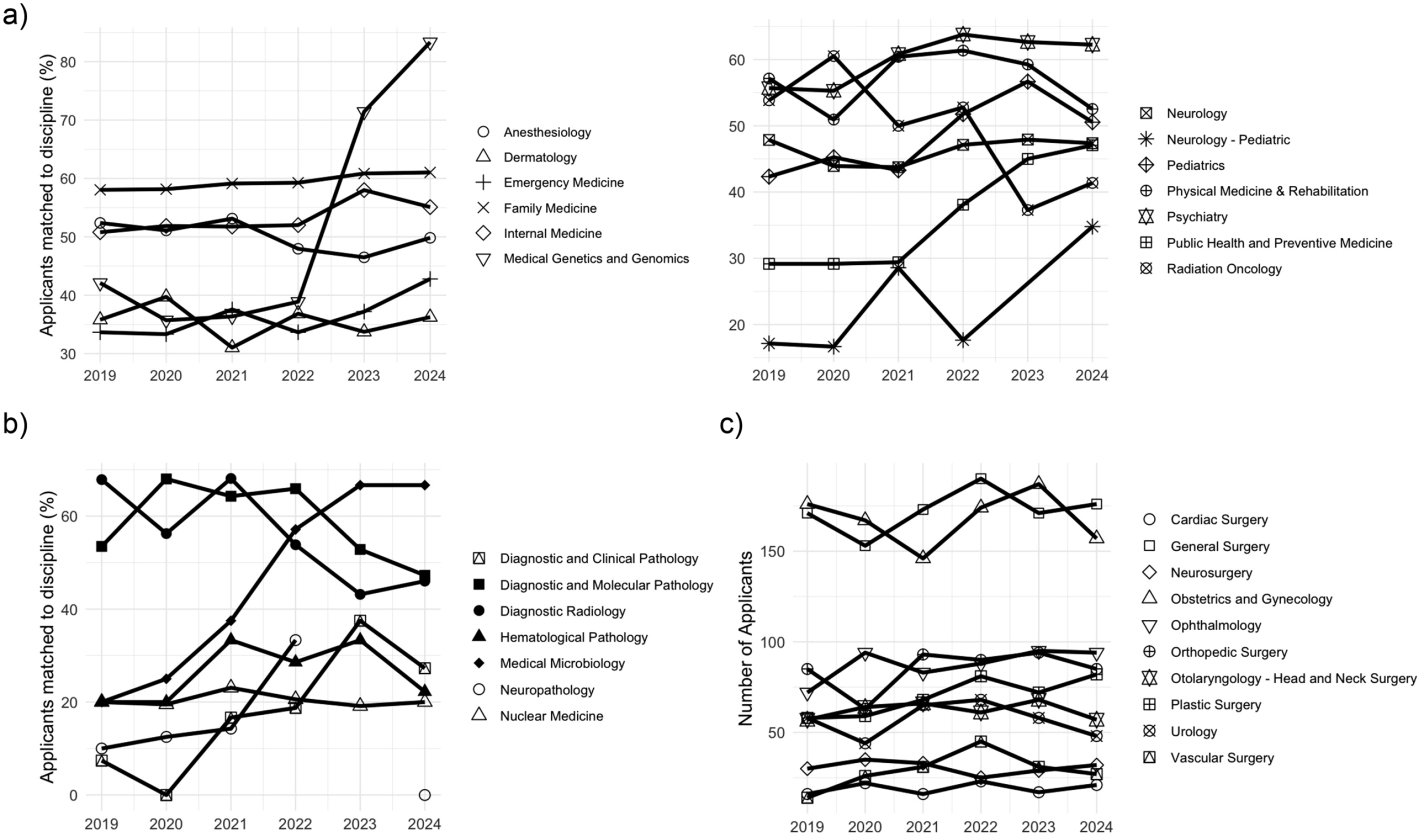
Number of applicants matched to each discipline grouped by (a) clinical disciplines, (b) diagnostic disciplines, and (c) surgical disciplines.

Within the clinical discipline cluster, family medicine (r2 = 0.964, p < 0.01), medical genetics (r2 = 0.817, p = 0.04), psychiatry (r2 = 0.839, p = 0.04), and public health (r2 = 0.939, p < 0.01) experienced a significant increase in match rate. Six specialties exhibited a positive trend in match rate (medical genetics and genomics, emergency medicine, psychiatry, public health and preventive medicine, pediatrics, pediatric neurology). Two specialties exhibited a negative trend in match rate (radiation oncology, physical medicine and rehabilitation). From 2022 to 2024, medical genetics and genomics experienced a 45% increase in match rate. On the other hand, from 2020 to 2024, radiation oncology experienced a 19% decrease in match rate (Fig 3a, S3 Table).

Within the diagnostic discipline cluster, diagnostic and clinical pathology (r2 = 0.850, p = 0.03) and medical microbiology (r2 = 0.971, p < 0.01) experienced a significant increase in match rate while diagnostic radiology (r2 = −0.825, p = 0.04) experienced a significant decrease in match rate. Two specialties exhibited a positive trend in match rate (medical microbiology, diagnostic and clinical pathology), two specialties exhibited a negative trend in match rate (diagnostic and molecular pathology, diagnostic radiology), and two specialties exhibited a relatively stable trend in match rate (nuclear medicine, hematological pathology). Neuropathology did not matriculate any students in 2024. From 2019 to 2024, medical microbiology experienced a 46% increase in match rate (Fig 3b, S3 Table).

Within the surgical discipline cluster, no specialties experienced a significant change in match rate. Three specialties exhibited a negative parabolic trend from 2019 to 2024 (vascular surgery, urology, otolaryngology), while neurosurgery exhibited a positive parabolic trend. Two specialties maintained a relatively stable match rate (general surgery, ophthalmology). Cardiac surgery and orthopedic surgery appeared to swing between increased and decreased match rates from year to year. Obstetrics and gynecology exhibited a positive trend while plastic surgery exhibited a negative trend (Fig 3c, S3 Table).

### 3.4 Q4: What are the gender differences in match outcomes by specialty?

In terms of gender-specific first choice match outcome, from 2019 to 2024, the number of females who matched to their first choice discipline increased from 1308 to 1493 while the number of males who matched to their first choice discipline decreased from 1063 to 971.

**Fig 4:**
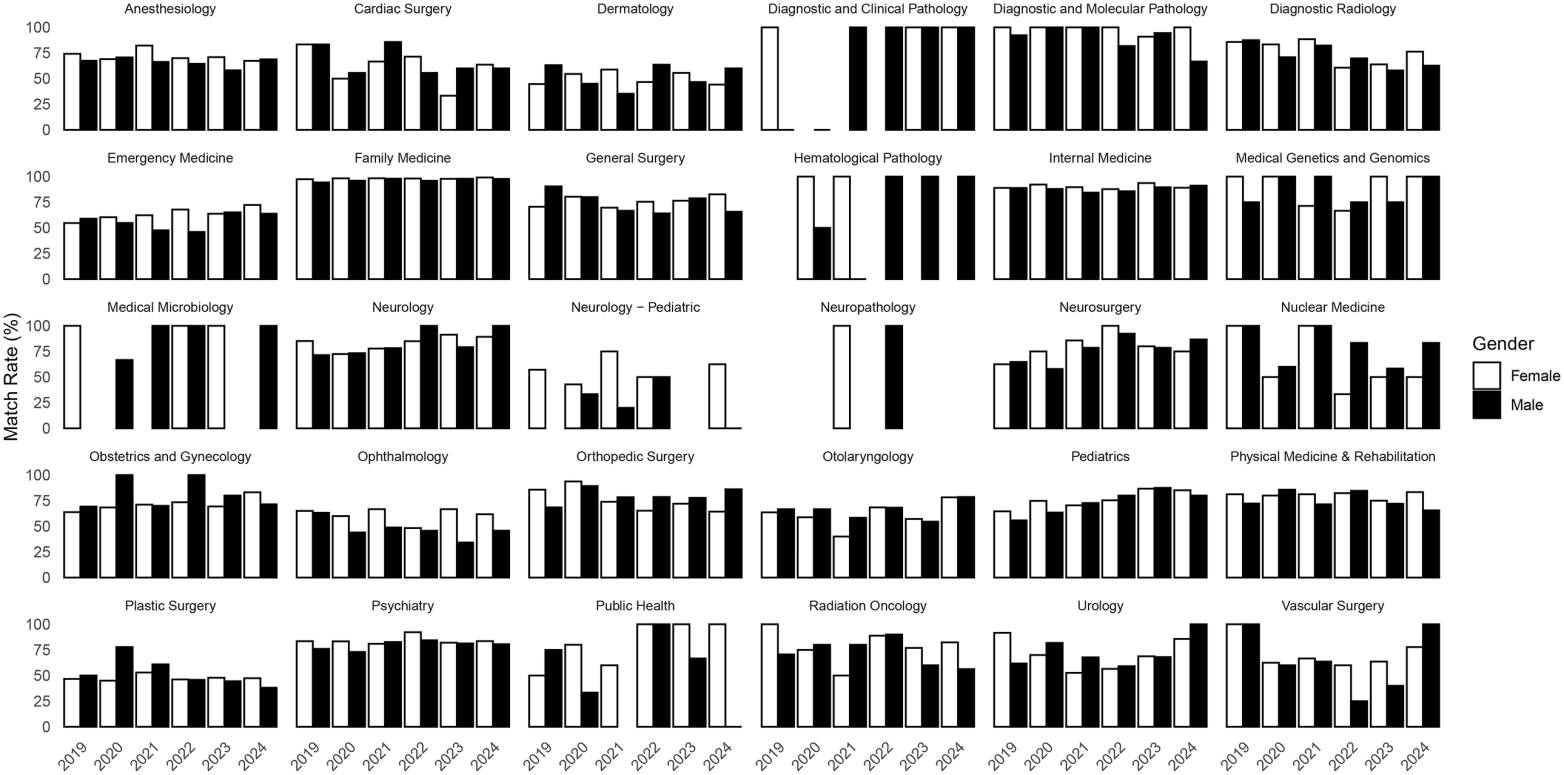
Gender differences in first choice match outcomes.

In Fig 4, the match rate trends for most specialties were similar for both genders. Of note, from 2019 to 2024, ophthalmology demonstrated a higher match rate for female applicants (average of 14.5% difference). Pediatric neurology and anesthesiology had female match rates either equal to or higher than male applicants. From 2021 to 2024, orthopedic surgery demonstrated a higher match rate for male applicants (average of 11.1% difference). Nuclear medicine had male match rates either equal to or higher than female applicants. Family medicine consistently had over 90% first choice match rate for both genders, while plastic surgery mostly had under 50% first choice match rate for both genders (Fig 4, S4 Table).

## 4. Discussion

The trend in CaRMS applications among Canadian Medical Graduates (CMGs) from 2019 to 2024 reflects a complex dynamic between applicant interest and available residency positions. Despite an overall decline in the number of CMGs applying to CaRMS over this period, the total quota of positions allocated for CMGs has increased. This development suggests ongoing efforts to expand training opportunities, potentially in response to workforce demands or policy changes. Notably, family medicine saw a steady decline in applicants from 2019 to 2023, aligning with broader concerns regarding the specialty’s perceived challenges, including workload and compensation. However, the reversal in 2024, with 91 more applicants than in 2023, may indicate renewed interest in the discipline, possibly influenced by recent initiatives to enhance recruitment or address primary care shortages. For instance, recent changes in payment models aimed at attracting family physicians, such as the Longitudinal Family Physician model in 2023, may contribute to an increase in interest [6]. Further investigation is needed to understand whether this increase represents a sustained shift or a temporary fluctuation in application patterns. Shifting political priorities in Canada, including changes to healthcare funding, medical school expansion, and workforce planning, may indirectly influence specialty application patterns. While CaRMS data do not capture these contextual factors, future research that integrates policy analyses with residency match trends could provide valuable insights into how government decisions shape medical students’ specialty choices.

The increasing proportion of female applicants to CaRMS highlights shifting gender dynamics in medical specialty preferences. This trend was particularly evident in many clinical disciplines, where female applicants consistently outnumbered male applicants across all six years. Dermatology, family medicine, pediatrics, and psychiatry were among the specialties with higher female representation, with dermatology and pediatrics seeing figures of 74% and 87% female applicants, respectively, in 2024. Surgical specialties, particularly general surgery and obstetrics and gynecology, also exhibited increasing female representation, with the gender gap widening each year. Ophthalmology showed a notable increase in female applicants, rising from 34.5% in 2019 to 49.3% in 2024. In contrast, specialties such as anesthesiology, diagnostic radiology, neurosurgery, orthopedic surgery, and physical medicine and rehabilitation continued to attract more male applicants. These trends suggest evolving gender preferences in medical specialties, possibly influenced by factors such as work-life balance, lifestyle considerations, and changing societal expectations [8].

Several clinical specialties, including medical genetics and genomics, emergency medicine, psychiatry, public health, pediatrics, and pediatric neurology, saw increased first-choice match rates, with the most significant increases in medical genetics and genomics and medical microbiology. These increases may reflect more available residency spots rather than a rise in applicants, and further analysis is needed to determine whether the pandemic influenced these trends. Specialties with decreased match rates included radiation oncology, physical medicine and rehabilitation, diagnostic and molecular pathology, diagnostic radiology, vascular surgery, urology, and otolaryngology. Family medicine, psychiatry, and neurosurgery maintained consistently high match rates (over 60% in 2023 and 2024), while dermatology, pathology, hematological pathology, nuclear medicine, and plastic surgery had the lowest match rates (under 40%). It is important to note that these match rates reflect first-choice specialty matches but not first-choice location matches, suggesting a need for further data on applicants’ location preferences.

Overall, most specialties displayed similar match rates for female and male applicants. Increased efforts to improve diversity is evident with the 2021 implementation of CaRMS self-identification questionnaire [5]. However, there were notable exceptions. In ophthalmology, female applicants had a higher match rate compared to their male counterparts. On the other hand, orthopedic surgery showed a higher match rate for male applicants. These exceptions highlight that while gender parity in match rates is largely evident, certain specialties still exhibit differences that warrant further exploration to understand the underlying factors contributing to these variations.

### 4.1 Limitations and future directions

This study has several limitations. Firstly, the analysis was restricted to first-choice specialty data, which does not account for the total number of gender-specific applications to each specialty. This method may have introduced bias, as applicants may list multiple specialties in their application, reflecting broader interests. Secondly, gender X was excluded from the analysis due to a small sample size, limiting the generalizability of the findings to this group. Additionally, IMGs were excluded, which restricts the applicability of our results to Canadian medical graduates only. Finally, the study focused exclusively on the first iteration of applications, potentially overlooking trends or preferences evident in subsequent iterations. Analyses using first-choice discipline match rate may not fully capture other factors that candidates consider important. For instance, some applicants may prioritize securing a position in their preferred training location over their chosen discipline, while others may find their second-choice discipline to be an equally acceptable outcome. The influence of couples match on specialty selection and match outcomes also could not be assessed due to the lack of publicly available data and represents an important area for future investigation. These factors should be considered when interpreting the findings and their implications.

Future research should aim to build on these findings by incorporating broader applicant populations, including international medical graduates and gender “X,” as well as data from second-iteration matches to capture a more complete picture of residency selection dynamics. Examining geographic match patterns and incorporating diversity metrics beyond gender could provide additional insight into equity and representation across specialties. Furthermore, integrating qualitative approaches, such as surveys or interviews with applicants, would help to contextualize quantitative trends by exploring the motivations, perceptions, and program-level factors that shape specialty choice. Finally, multivariate analyses that combine applicant demographics, specialty characteristics, and workforce factors could better identify the drivers of match success and evolving specialty interest in Canada.

## 5. Conclusion

This retrospective analysis of specialty match rates and gender trends in Canadian residency applications from 2019 to 2024 highlights significant shifts that may have been influenced by the COVID-19 pandemic, evolving societal priorities, and advancements in medicine. The growing focus on physician wellness and awareness of burnout has likely shaped applicant preferences, alongside the increasing integration of artificial intelligence and digital technologies in medical practice. Moreover, the push for equity, diversity, and inclusion initiatives has emphasized the importance of fostering a more representative healthcare workforce. These findings underscore the need for ongoing monitoring of application trends to ensure alignment with the dynamic landscape of healthcare and the diverse needs of patients and providers.

## Supporting information

S1 Table Total number of applications to each specialty. (DOCX)

S1 Fig Specialty competitiveness grouped by (a) clinical disciplines, (b) surgical disciplines, and (c) diagnostic disciplines. (DOCX)

S2 Table Proportion of applications by gender to each discipline. (DOCX)

S3 Table Number of applicants matched to each discipline. (DOCX)

S4 Table Gender differences in first choice match outcomes. (DOCX)
