# Supplementary material for: A retrospective analysis of specialty match rate and gender trends in Canadian residency applications (2019–2024)
Source: PLoS One. 2025 Oct 30;20(10):e0334134. doi: 10.1371/journal.pone.0334134 (PMC12574843; doi:10.1371/journal.pone.0334134)
Supplement: S1 Table — (DOCX) [file pone.0334134.s001.docx]

**S1 Table. Total number of applications to each specialty**

| **Specialty** | **2024** | **2023** | **2022** | **2021** | **2020** | **2019** |
| --- | --- | --- | --- | --- | --- | --- |
| Anesthesiology | 297 | 299 | 269 | 224 | 227 | 210 |
| Cardiac Surgery | 21 | 17 | 23 | 16 | 22 | 16 |
| Dermatology | 80 | 83 | 76 | 87 | 73 | 81 |
| Diagnostic Radiology | 176 | 183 | 143 | 116 | 144 | 112 |
| Diagnostic and Clinical Pathology | 11 | 16 | 16 | 12 | 15 | 27 |
| Diagnostic and Molecular Pathology | 55 | 53 | 44 | 42 | 50 | 43 |
| Emergency Medicine | 194 | 207 | 208 | 189 | 219 | 208 |
| Family Medicine | 1999 | 1908 | 1991 | 2021 | 2125 | 2150 |
| General Surgery | 176 | 171 | 190 | 173 | 153 | 171 |
| Hematological Pathology | 9 | 6 | 7 | 9 | 10 | 5 |
| Internal Medicine | 864 | 793 | 869 | 881 | 885 | 886 |
| Medical Genetics and Genomics | 12 | 14 | 18 | 22 | 14 | 19 |
| Medical Microbiology | 3 | 3 | 7 | 8 | 12 | 5 |
| Neurology | 95 | 96 | 87 | 112 | 107 | 94 |
| Neurology - Pediatric | 23 |  | 17 | 28 | 48 | 35 |
| Neuropathology | 3 | 0 | 6 | 7 | 8 | 10 |
| Neurosurgery | 32 | 29 | 25 | 33 | 35 | 30 |
| Nuclear Medicine | 45 | 47 | 34 | 26 | 41 | 30 |
| Obstetrics and Gynecology | 157 | 187 | 174 | 146 | 167 | 176 |
| Ophthalmology | 94 | 95 | 88 | 83 | 94 | 72 |
| Orthopedic Surgery | 85 | 94 | 90 | 93 | 63 | 85 |
| Otolaryngology - Head and Neck Surgery | 57 | 68 | 61 | 66 | 64 | 57 |
| Pediatrics | 273 | 247 | 255 | 298 | 305 | 300 |
| Physical Medicine & Rehabilitation | 59 | 54 | 44 | 48 | 53 | 49 |
| Plastic Surgery | 82 | 72 | 81 | 68 | 59 | 58 |
| Psychiatry | 294 | 257 | 265 | 291 | 331 | 316 |
| Public Health and Preventive Medicine | 17 | 20 | 21 | 17 | 24 | 24 |
| Radiation Oncology | 58 | 59 | 36 | 36 | 38 | 39 |
| Urology | 48 | 58 | 68 | 65 | 44 | 58 |
| Vascular Surgery | 27 | 31 | 45 | 31 | 26 | 14 |
