# Supplementary material for: A retrospective analysis of specialty match rate and gender trends in Canadian residency applications (2019–2024)
Source: PLoS One. 2025 Oct 30;20(10):e0334134. doi: 10.1371/journal.pone.0334134 (PMC12574843; doi:10.1371/journal.pone.0334134)
Supplement: S1 Fig — (DOCX) [file pone.0334134.s002.docx]

**
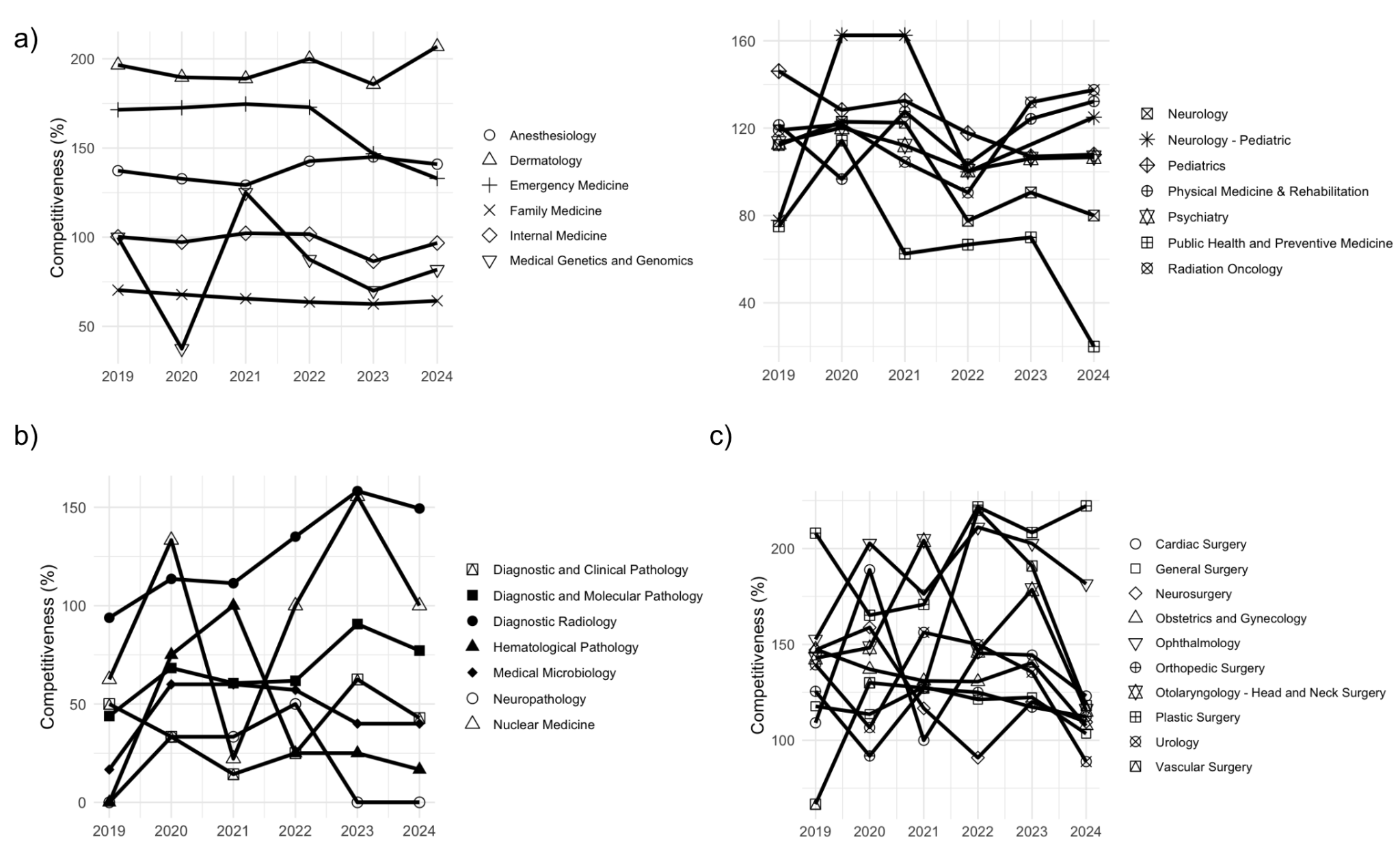
**

**S1 Figure. Specialty competitiveness grouped by (a) clinical disciplines, (b) surgical disciplines, and (c) diagnostic disciplines.**
