# Supplementary material for: A retrospective analysis of specialty match rate and gender trends in Canadian residency applications (2019–2024)
Source: PLoS One. 2025 Oct 30;20(10):e0334134. doi: 10.1371/journal.pone.0334134 (PMC12574843; doi:10.1371/journal.pone.0334134)
Supplement: S2 Table — (DOCX) [file pone.0334134.s003.docx]

**S2 Table. Proportion of applications by gender to each discipline**

| **Specialty** | **2024** | | **2023** | | **2022** | | **2021** | | **2020** | | **2019** | |
| --- | --- | --- | --- | --- | --- | --- | --- | --- | --- | --- | --- | --- |
|  | **Female** | **Male** | **Female** | **Male** | **Female** | **Male** | **Female** | **Male** | **Female** | **Male** | **Female** | **Male** |
| Anesthesiology | 101 (48.1%) | 109 (51.9%) | 86 (42.4%) | 117 (57.6%) | 80 (43.5%) | 104 (56.5%) | 62 (40.3%) | 92 (59.7%) | 71 (46.4%) | 82 (53.6%) | 62 (41.1%) | 89 (58.9%) |
| Cardiac Surgery | 11 (68.8%) | 5 (31.2%) | 3 (23.1%) | 10 (76.9%) | 7 (43.8%) | 9 (56.2%) | 3 (30%) | 7 (70%) | 8 (47.1%) | 9 (52.9%) | 6 (50%) | 6 (50%) |
| Dermatology | 43 (74.1%) | 15 (25.9%) | 36 (70.6%) | 15 (29.4%) | 45 (80.4%) | 11 (19.6%) | 34 (66.7%) | 17 (33.3%) | 33 (62.3%) | 20 (37.7%) | 38 (66.7%) | 19 (33.3%) |
| Diagnostic Radiology | 38 (31.4%) | 83 (68.6%) | 36 (29%) | 88 (71%) | 28 (26.9%) | 76 (73.1%) | 26 (29.5%) | 62 (70.5%) | 36 (39.6%) | 55 (60.4%) | 28 (36.8%) | 48 (63.2%) |
| Diagnostic and Clinical Pathology | 1 (33.3%) | 2 (66.7%) | 2 (40%) | 3 (60%) | 0 (0%) | 1 (100%) | 0 (0%) | 1 (100%) | 0 (0%) | 2 (100%) | 2 (66.7%) | 1 (33.3%) |
| Diagnostic and Molecular Pathology | 12 (44.4%) | 15 (55.6%) | 11 (37.9%) | 18 (62.1%) | 10 (47.6%) | 11 (52.4%) | 13 (65%) | 7 (35%) | 14 (50%) | 14 (50%) | 5 (27.8%) | 13 (72.2%) |
| Emergency Medicine | 65 (58%) | 47 (42%) | 69 (61.6%) | 43 (38.4%) | 56 (47.1%) | 63 (52.9%) | 61 (49.2%) | 63 (50.8%) | 53 (42.1%) | 73 (57.9%) | 64 (53.3%) | 56 (46.7%) |
| Family Medicine | 584 (62.7%) | 347 (37.3%) | 550 (62.3%) | 333 (37.7%) | 537 (61%) | 344 (39%) | 556 (61.7%) | 345 (38.3%) | 590 (62.8%) | 350 (37.2%) | 588 (60.4%) | 386 (39.6%) |
| General Surgery | 52 (59.8%) | 35 (40.2%) | 68 (67.3%) | 33 (32.7%) | 57 (59.4%) | 39 (40.6%) | 66 (66.7%) | 33 (33.3%) | 56 (61.5%) | 35 (38.5%) | 51 (54.8%) | 42 (45.2%) |
| Hematological Pathology | 0 (0%) | 1 (100%) | 0 (0%) | 1 (100%) | 0 (0%) | 1 (100%) | 3 (75%) | 1 (25%) | 1 (33.3%) | 2 (66.7%) | 0 (NaN%) | 0 (NaN%) |
| Internal Medicine | 258 (54.5%) | 215 (45.5%) | 206 (50.5%) | 202 (49.5%) | 229 (49.8%) | 231 (50.2%) | 223 (47.9%) | 243 (52.1%) | 232 (51.8%) | 216 (48.2%) | 199 (43.3%) | 261 (56.7%) |
| Medical Genetics and Genomics | 6 (66.7%) | 3 (33.3%) | 3 (42.9%) | 4 (57.1%) | 3 (42.9%) | 4 (57.1%) | 7 (70%) | 3 (30%) | 2 (66.7%) | 1 (33.3%) | 4 (50%) | 4 (50%) |
| Medical Microbiology | 0 (0%) | 2 (100%) | 2 (100%) | 0 (0%) | 3 (75%) | 1 (25%) | 0 (0%) | 3 (100%) | 0 (0%) | 3 (100%) | 1 (100%) | 0 (0%) |
| Neurology | 28 (68.3%) | 13 (31.7%) | 23 (48.9%) | 24 (51.1%) | 20 (52.6%) | 18 (47.4%) | 36 (61%) | 23 (39%) | 29 (49.2%) | 30 (50.8%) | 27 (49.1%) | 28 (50.9%) |
| Neurology - Pediatric | 8 (88.9%) | 1 (11.1%) |  |  | 4 (66.7%) | 2 (33.3%) | 8 (61.5%) | 5 (38.5%) | 7 (53.8%) | 6 (46.2%) | 7 (100%) | 0 (0%) |
| Neuropathology | 0 (NaN%) | 0 (NaN%) | 0 (NaN%) | 0 (NaN%) | 0 (0%) | 2 (100%) | 1 (100%) | 0 (0%) | 0 (NaN%) | 0 (NaN%) | 0 (NaN%) | 0 (NaN%) |
| Neurosurgery | 8 (34.8%) | 15 (65.2%) | 10 (41.7%) | 14 (58.3%) | 7 (35%) | 13 (65%) | 7 (33.3%) | 14 (66.7%) | 8 (29.6%) | 19 (70.4%) | 8 (32%) | 17 (68%) |
| Nuclear Medicine | 4 (40%) | 6 (60%) | 2 (14.3%) | 12 (85.7%) | 3 (33.3%) | 6 (66.7%) | 1 (50%) | 1 (50%) | 2 (16.7%) | 10 (83.3%) | 4 (80%) | 1 (20%) |
| Obstetrics and Gynecology | 89 (92.7%) | 7 (7.3%) | 124 (96.1%) | 5 (3.9%) | 98 (89.1%) | 12 (10.9%) | 97 (90.7%) | 10 (9.3%) | 98 (90.7%) | 10 (9.3%) | 105 (89%) | 13 (11%) |
| Ophthalmology | 34 (49.3%) | 35 (50.7%) | 36 (46.8%) | 41 (53.2%) | 29 (38.7%) | 46 (61.3%) | 24 (35.8%) | 43 (64.2%) | 25 (33.3%) | 50 (66.7%) | 20 (34.5%) | 38 (65.5%) |
| Orthopedic Surgery | 28 (43.8%) | 36 (56.2%) | 25 (41%) | 36 (59%) | 23 (35.4%) | 42 (64.6%) | 23 (38.3%) | 37 (61.7%) | 16 (36.4%) | 28 (63.6%) | 21 (35.6%) | 38 (64.4%) |
| Otolaryngology - Head and Neck Surgery | 23 (62.2%) | 14 (37.8%) | 28 (56%) | 22 (44%) | 19 (46.3%) | 22 (53.7%) | 25 (51%) | 24 (49%) | 17 (41.5%) | 24 (58.5%) | 22 (55%) | 18 (45%) |
| Pediatrics | 128 (86.5%) | 20 (13.5%) | 128 (84.2%) | 24 (15.8%) | 134 (81.7%) | 30 (18.3%) | 142 (81.1%) | 33 (18.9%) | 135 (76.7%) | 41 (23.3%) | 144 (77%) | 43 (23%) |
| Physical Medicine & Rehabilitation | 12 (29.3%) | 29 (70.7%) | 16 (39%) | 25 (61%) | 17 (56.7%) | 13 (43.3%) | 16 (43.2%) | 21 (56.8%) | 15 (51.7%) | 14 (48.3%) | 16 (47.1%) | 18 (52.9%) |
| Plastic Surgery | 38 (64.4%) | 21 (35.6%) | 23 (46%) | 27 (54%) | 26 (52%) | 24 (48%) | 17 (42.5%) | 23 (57.5%) | 20 (52.6%) | 18 (47.4%) | 30 (57.7%) | 22 (42.3%) |
| Psychiatry | 134 (65%) | 72 (35%) | 117 (60.9%) | 75 (39.1%) | 103 (57.2%) | 77 (42.8%) | 121 (59.9%) | 81 (40.1%) | 114 (53.3%) | 100 (46.7%) | 109 (53.2%) | 96 (46.8%) |
| Public Health and Preventive Medicine | 1 (50%) | 1 (50%) | 4 (57.1%) | 3 (42.9%) | 5 (83.3%) | 1 (16.7%) | 5 (100%) | 0 (0%) | 5 (62.5%) | 3 (37.5%) | 2 (33.3%) | 4 (66.7%) |
| Radiation Oncology | 17 (51.5%) | 16 (48.5%) | 13 (46.4%) | 15 (53.6%) | 9 (47.4%) | 10 (52.6%) | 8 (34.8%) | 15 (65.2%) | 8 (28.6%) | 20 (71.4%) | 8 (32%) | 17 (68%) |
| Urology | 14 (43.8%) | 18 (56.2%) | 16 (39%) | 25 (61%) | 23 (51.1%) | 22 (48.9%) | 19 (38%) | 31 (62%) | 10 (31.2%) | 22 (68.8%) | 12 (26.1%) | 34 (73.9%) |
| Vascular Surgery | 9 (69.2%) | 4 (30.8%) | 11 (52.4%) | 10 (47.6%) | 10 (45.5%) | 12 (54.5%) | 3 (21.4%) | 11 (78.6%) | 8 (61.5%) | 5 (38.5%) | 4 (66.7%) | 2 (33.3%) |
