# Supplementary material for: A retrospective analysis of specialty match rate and gender trends in Canadian residency applications (2019–2024)
Source: PLoS One. 2025 Oct 30;20(10):e0334134. doi: 10.1371/journal.pone.0334134 (PMC12574843; doi:10.1371/journal.pone.0334134)
Supplement: S3 Table — (DOCX) [file pone.0334134.s004.docx]

**S3 Table. Number of applicants matched to each discipline**

| **Specialty** | **2024** | **2023** | **2022** | **2021** | **2020** | **2019** |
| --- | --- | --- | --- | --- | --- | --- |
| Anesthesiology | 49.8 % | 46.5 % | 48 % | 53.1 % | 51.1 % | 52.4 % |
| Cardiac Surgery | 47.6 % | 52.9 % | 47.8 % | 62.5 % | 40.9 % | 68.8 % |
| Dermatology | 36.2 % | 33.7 % | 36.8 % | 31 % | 39.7 % | 35.8 % |
| Diagnostic Radiology | 46 % | 43.2 % | 53.8 % | 68.1 % | 56.2 % | 67.9 % |
| Diagnostic and Clinical Pathology | 27.3 % | 37.5 % | 18.8 % | 16.7 % | 0 % | 7.4 % |
| Diagnostic and Molecular Pathology | 47.3 % | 52.8 % | 65.9 % | 64.3 % | 68 % | 53.5 % |
| Emergency Medicine | 42.8 % | 37.2 % | 33.7 % | 37.6 % | 33.3 % | 33.7 % |
| Family Medicine | 61 % | 60.8 % | 59.3 % | 59.1 % | 58.2 % | 58 % |
| General Surgery | 44.3 % | 49.7 % | 42.1 % | 45.7 % | 51 % | 46.2 % |
| Hematological Pathology | 22.2 % | 33.3 % | 28.6 % | 33.3 % | 20 % | 20 % |
| Internal Medicine | 55.1 % | 58 % | 52 % | 51.8 % | 51.9 % | 50.8 % |
| Medical Genetics and Genomics | 83.3 % | 71.4 % | 38.9 % | 36.4 % | 35.7 % | 42.1 % |
| Medical Microbiology | 66.7 % | 66.7 % | 57.1 % | 37.5 % | 25 % | 20 % |
| Neurology | 47.4 % | 47.9 % | 47.1 % | 43.8 % | 43.9 % | 47.9 % |
| Neurology - Pediatric | 34.8 % |  | 17.6 % | 28.6 % | 16.7 % | 17.1 % |
| Neuropathology | 0 % |  | 33.3 % | 14.3 % | 12.5 % | 10 % |
| Neurosurgery | 62.5 % | 69 % | 76 % | 54.5 % | 48.6 % | 56.7 % |
| Nuclear Medicine | 20 % | 19.1 % | 20.6 % | 23.1 % | 19.5 % | 20 % |
| Obstetrics and Gynecology | 54.8 % | 50.3 % | 48.3 % | 57.5 % | 47.9 % | 45.5 % |
| Ophthalmology | 40.4 % | 40 % | 40.9 % | 45.8 % | 39.4 % | 52.8 % |
| Orthopedic Surgery | 65.9 % | 55.3 % | 57.8 % | 51.6 % | 69.8 % | 55.3 % |
| Otolaryngology - Head and Neck Surgery | 56.1 % | 41.2 % | 45.9 % | 36.4 % | 45.3 % | 49.1 % |
| Pediatrics | 50.5 % | 56.7 % | 51.8 % | 43.3 % | 45.2 % | 42.3 % |
| Physical Medicine & Rehabilitation | 52.5 % | 59.3 % | 61.4 % | 60.4 % | 50.9 % | 57.1 % |
| Plastic Surgery | 32.9 % | 33.3 % | 28.4 % | 35.3 % | 39 % | 43.1 % |
| Psychiatry | 62.2 % | 62.6 % | 63.8 % | 60.8 % | 55.3 % | 55.7 % |
| Public Health and Preventive Medicine | 47.1 % | 45 % | 38.1 % | 29.4 % | 29.2 % | 29.2 % |
| Radiation Oncology | 41.4 % | 37.3 % | 52.8 % | 50 % | 60.5 % | 53.8 % |
| Urology | 70.8 % | 53.4 % | 44.1 % | 49.2 % | 63.6 % | 56.9 % |
| Vascular Surgery | 40.7 % | 35.5 % | 22.2 % | 32.3 % | 34.6 % | 50 % |
