# Supplementary material for: A retrospective analysis of specialty match rate and gender trends in Canadian residency applications (2019–2024)
Source: PLoS One. 2025 Oct 30;20(10):e0334134. doi: 10.1371/journal.pone.0334134 (PMC12574843; doi:10.1371/journal.pone.0334134)
Supplement: S4 Table — (DOCX) [file pone.0334134.s005.docx]

**S4 Table. Gender differences in first choice match outcomes**

| **Specialty** | **2024** | | **2023** | | **2022** | | **2021** | | **2020** | | **2019** | |
| --- | --- | --- | --- | --- | --- | --- | --- | --- | --- | --- | --- | --- |
|  | **Female** | **Male** | **Female** | **Male** | **Female** | **Male** | **Female** | **Male** | **Female** | **Male** | **Female** | **Male** |
| Anesthesiology | 67.3 % | 68.8 % | 70.9 % | 58.1 % | 70 % | 64.4 % | 82.3 % | 66.3 % | 69 % | 70.7 % | 74.2 % | 67.4 % |
| Cardiac Surgery | 63.6 % | 60 % | 33.3 % | 60 % | 71.4 % | 55.6 % | 66.7 % | 85.7 % | 50 % | 55.6 % | 83.3 % | 83.3 % |
| Dermatology | 44.2 % | 60 % | 55.6 % | 46.7 % | 46.7 % | 63.6 % | 58.8 % | 35.3 % | 54.5 % | 45 % | 44.7 % | 63.2 % |
| Diagnostic Radiology | 76.3 % | 62.7 % | 63.9 % | 58 % | 60.7 % | 69.7 % | 88.5 % | 82.3 % | 83.3 % | 70.9 % | 85.7 % | 87.5 % |
| Diagnostic and Clinical Pathology | 100 % | 100 % | 100 % | 100 % | NaN % | 100 % | NaN % | 100 % | NaN % | 0 % | 100 % | 0 % |
| Diagnostic and Molecular Pathology | 100 % | 66.7 % | 90.9 % | 94.4 % | 100 % | 81.8 % | 100 % | 100 % | 100 % | 100 % | 100 % | 92.3 % |
| Emergency Medicine | 72.3 % | 63.8 % | 63.8 % | 65.1 % | 67.9 % | 46 % | 62.3 % | 47.6 % | 60.4 % | 54.8 % | 54.7 % | 58.9 % |
| Family Medicine | 99.1 % | 97.7 % | 97.8 % | 97.9 % | 98.1 % | 95.9 % | 98.4 % | 98 % | 98.3 % | 96 % | 97.4 % | 94.3 % |
| General Surgery | 82.7 % | 65.7 % | 76.5 % | 78.8 % | 75.4 % | 64.1 % | 69.7 % | 66.7 % | 80.4 % | 80 % | 70.6 % | 90.5 % |
| Hematological Pathology | NaN % | 100 % | NaN % | 100 % | NaN % | 100 % | 100 % | 0 % | 100 % | 50 % | NaN % | NaN % |
| Internal Medicine | 89.1 % | 91.2 % | 93.7 % | 89.6 % | 87.8 % | 85.7 % | 89.7 % | 84.4 % | 92.2 % | 88 % | 88.9 % | 88.9 % |
| Medical Genetics and Genomics | 100 % | 100 % | 100 % | 75 % | 66.7 % | 75 % | 71.4 % | 100 % | 100 % | 100 % | 100 % | 75 % |
| Medical Microbiology | NaN % | 100 % | 100 % | NaN % | 100 % | 100 % | NaN % | 100 % | NaN % | 66.7 % | 100 % | NaN % |
| Neurology | 89.3 % | 100 % | 91.3 % | 79.2 % | 85 % | 100 % | 77.8 % | 78.3 % | 72.4 % | 73.3 % | 85.2 % | 71.4 % |
| Neurology - Pediatric | 62.5 % | 0 % |  |  | 50 % | 50 % | 75 % | 20 % | 42.9 % | 33.3 % | 57.1 % | NaN % |
| Neuropathology | NaN % | NaN % | NaN % | NaN % | NaN % | 100 % | 100 % | NaN % | NaN % | NaN % | NaN % | NaN % |
| Neurosurgery | 75 % | 86.7 % | 80 % | 78.6 % | 100 % | 92.3 % | 85.7 % | 78.6 % | 75 % | 57.9 % | 62.5 % | 64.7 % |
| Nuclear Medicine | 50 % | 83.3 % | 50 % | 58.3 % | 33.3 % | 83.3 % | 100 % | 100 % | 50 % | 60 % | 100 % | 100 % |
| Obstetrics and Gynecology | 83.1 % | 71.4 % | 69.4 % | 80 % | 73.5 % | 100 % | 71.1 % | 70 % | 68.4 % | 100 % | 63.8 % | 69.2 % |
| Ophthalmology | 61.8 % | 45.7 % | 66.7 % | 34.1 % | 48.3 % | 45.7 % | 66.7 % | 48.8 % | 60 % | 44 % | 65 % | 63.2 % |
| Orthopedic Surgery | 64.3 % | 86.1 % | 72 % | 77.8 % | 65.2 % | 78.6 % | 73.9 % | 78.4 % | 93.8 % | 89.3 % | 85.7 % | 68.4 % |
| Otolaryngology - Head and Neck Surgery | 78.3 % | 78.6 % | 57.1 % | 54.5 % | 68.4 % | 68.2 % | 40 % | 58.3 % | 58.8 % | 66.7 % | 63.6 % | 66.7 % |
| Pediatrics | 85.2 % | 80 % | 86.7 % | 87.5 % | 75.4 % | 80 % | 70.4 % | 72.7 % | 74.8 % | 63.4 % | 64.6 % | 55.8 % |
| Physical Medicine & Rehabilitation | 83.3 % | 65.5 % | 75 % | 72 % | 82.4 % | 84.6 % | 81.2 % | 71.4 % | 80 % | 85.7 % | 81.2 % | 72.2 % |
| Plastic Surgery | 47.4 % | 38.1 % | 47.8 % | 44.4 % | 46.2 % | 45.8 % | 52.9 % | 60.9 % | 45 % | 77.8 % | 46.7 % | 50 % |
| Psychiatry | 83.6 % | 80.6 % | 82.1 % | 81.3 % | 92.2 % | 84.4 % | 81 % | 82.7 % | 83.3 % | 73 % | 83.5 % | 76 % |
| Public Health and Preventive Medicine | 100 % | 0 % | 100 % | 66.7 % | 100 % | 100 % | 60 % | NaN % | 80 % | 33.3 % | 50 % | 75 % |
| Radiation Oncology | 82.4 % | 56.2 % | 76.9 % | 60 % | 88.9 % | 90 % | 50 % | 80 % | 75 % | 80 % | 100 % | 70.6 % |
| Urology | 85.7 % | 100 % | 68.8 % | 68 % | 56.5 % | 59.1 % | 52.6 % | 67.7 % | 70 % | 81.8 % | 91.7 % | 61.8 % |
| Vascular Surgery | 77.8 % | 100 % | 63.6 % | 40 % | 60 % | 25 % | 66.7 % | 63.6 % | 62.5 % | 60 % | 100 % | 100 % |
